# Could Alcohol Abuse Drive Intimate Partner Violence Perpetrators’ Psychophysiological Response to Acute Stress?

**DOI:** 10.3390/ijerph15122729

**Published:** 2018-12-03

**Authors:** Sara Vitoria-Estruch, Ángel Romero-Martínez, Marisol Lila, Luis Moya-Albiol

**Affiliations:** 1 Department of Psychobiology, University of Valencia, 46010 Valencia, Spain; Sara.Vitoria@uv.es (S.V.-E.); Luis.Moya@uv.es (L.M.-A.); 2 Department of Social Psychology, University of Valencia, 46010 Valencia, Spain; Marisol.Lila@uv.es

**Keywords:** acute stress, cardiorespiratory variables, impulsivity, intimate partner violence, skin conductance, autonomic nervous system

## Abstract

Proactively aggressive individuals have been shown to present a different pattern of autonomic nervous system (ANS) dysregulation from that of individuals characterized by reactive violence. Although attempts have been made to classify intimate partner violence (IPV) perpetrators based on ANS reactivity to acute stress, subsequent studies have failed to replicate this classification. Notably, the proposed classification neglected the role of chronic alcohol abuse in ANS dysregulation and the fact that this dysregulation entails an abnormal stress response. The aim of the present study was to analyze the response profile (psychological state and ANS response) of groups of IPV perpetrators with high (*n* = 27) and low (*n* = 33)-risk alcohol use to an acute stressor, compared to controls (*n* = 35). All IPV perpetrators scored higher on executive dysfunctions and impulsivity and showed larger decreases in positive affect, less satisfaction, and a higher external locus of control after the stressor than controls. IPV perpetrators with low-risk alcohol use had higher skin conductance levels and breathing reactivity than controls, especially during preparatory, task, and recovery periods. This information could help to develop methods for increasing batterers’ behavioral self-regulation, thus decreasing IPV recidivism risk.

## 1. Introduction

Scientifically strong evidence has shown that autonomic nervous system (ANS) functioning can be employed as a classification criterion in violent men because perpetrators of proactive and reactive violence present a different pattern of autonomic dysregulation [1,2,3,4,5,6,7,8,9]. Nevertheless, it is not clear whether resting values of ANS measures accurately reflect proneness to violence. Therefore, it would be advisable to consider resting values as well as ANS reactivity to stress and/or specific stimuli in order to characterize the profile of generally violent individuals.

Regarding intimate partner violence (IPV), Gottman, Jacobson, Rushe, and Shortt [10] found evidence supporting two different IPV perpetrator categories, based on batterers’ psychophysiological reactivity to an acute laboratory stressor (marital conflict). On the one hand, these batterers were classified as Type I if they showed heart rate (HR) hypoactivity in confronting this type of stressor. In fact, Gottman et al. [10] interpreted batterers with this profile as psychopathic because they presented proactive violence and were more violent than the other batterers. Furthermore, IPV perpetrators of this type tend to employ manipulative strategies to control their wives. On the other hand, IPV perpetrators were classified as Type II if they presented HR hyperreactivity to stress. Additionally, they scored higher on dependent personality traits, and they usually employed impulsive/reactive violence. Nevertheless, two later studies by Babcock, Green, Webb, and Graham [11] and Meehan, Holtzworth-Munroe, and Herron [12] failed to replicate this classification. Although both studies employed similar methodologies (HR and psychological measurements and a laboratory stressor) as in Gottman et al. [10], the authors attributed their failure to replicate earlier findings to a methodological weakness in the initial study that interfered with the HR reactivity calculation. Specifically, Gottman et al. [10] measured HR resting values over a very short period of time, whereas the later studies used longer resting times in order to increase the reliability of the measurement and more accurately adjust for baseline in their analysis of the psychophysiological response to stress. Notably, the later studies did not find any significant differences in resting values or reactivity to stress between the two groups of IPV perpetrators.

Studies by Romero-Martínez et al. [5,6] have attempted to build on the results of Gottman et al. [10] by employing several cardiovascular and electrodermal markers that were not employed in previous studies in this field of research. Specifically, they compared the ANS response to a modified version of the Trier Social Stress Test (TSST) in IPV perpetrators described as reactive, based on their criminal record and psychological characteristics, and non-violent men. In the procedure, participants had to make a speech about their own experiences and problems with IPV and give their opinions about Spanish legislation, followed by a mental arithmetic test. In this study, IPV perpetrators showed an increase in their skin conductance level (hyperreactivity) when they prepared to confront the stress after researchers had presented the task instructions (preparatory period), and this hyperreactivity was associated with impulsivity traits. In addition, they had higher HRs, lower vagal ratios, and higher non-specific skin conductance responses (NSCRs) after the stressor ended (recovery period) than controls. Finally, they showed shorter pre-ejection period (PEP; higher sympathetic predominance) than controls throughout the assessment.

The sympathetic predominance observed may be indicative of ANS dysregulation. In this regard, individuals with this psychophysiological profile maintain high levels of vigilance (or activation), irritability, and tension (negative affect) over sustained periods of time. This dysregulation could reduce the threshold for violent behavior when exposed to certain types of stimuli that are incongruent with their hostile cognitive schemas, such as sexist ideas about women or dominant roles in relationships [13]. Additionally, the facilitation of violence might also be explained by IPV perpetrators’ cognitive processing deficits, which may include low processing speed and poor attention switching and sustained attention, as well as deficits in working memory and other impairments associated with executive dysfunctions, such as poor cognitive flexibility, planning abilities, and inhibitory control [14,15,16,17,18].

Gottman et al. [10] and later studies [11,12] neglected the role of chronic alcohol abuse in the development of cognitive impairments [16,17,18,19,20,21,22,23] and ANS dysregulation [24,25,26] in this kind of population, as well as the fact that this dysregulation entails an abnormal stress response. In fact, it has been suggested that chronic alcohol consumption tends to depress the central nervous system, suppressing excitatory nerve pathway activity in the resting state [27], but there are inconsistencies about whether alcohol tends to reduce sympathetic or parasympathetic control of the ANS [28,29,30,31,32,33]. Thus, it makes sense to study how alcohol disrupts IPV perpetrators’ response to stress.

The present study sought to confirm and extend the results of Romero-Martínez et al. [5,6], while including some changes in the experimental procedure and increasing the sample size, in order to improve our understanding of the complex phenomenon of IPV. Specifically, the first objective of this study was to analyze reactive IPV perpetrators’ psychological (trait and state) and physiological responses to a set of cognitive tests, namely, an acute laboratory stressor previously shown to produce psychophysiological activation [34,35], compared to a non-violent group (controls). Based on the results of Romero-Martínez et al. [5,6], we expected that reactive IPV perpetrators would present higher sympathetic predominance and lower vagal regulation in response to acute stress than controls. Moreover, an additional group of IPV perpetrators with high-risk alcohol use was included to compare their ANS response to that of the low-risk alcohol use IPV perpetrators and the non-violent group. Because chronic heavy alcohol consumption has a depressive effect on ANS activity and is associated with higher levels of the impulsivity trait [28,29,30,31,32,33], we hypothesized that IPV perpetrators who were heavy drinkers would, due to the effects of alcohol, show lower sympathetic predominance and higher vagal regulation in response to stress than IPV perpetrators with low alcohol consumption. This type of research seeks to help us improve our understanding of emotional and psychophysiological dysregulation in IPV perpetrators, which may underlie their predisposition to violence.

## 2. Materials and Methods

### 2.1. Participants

The final sample was composed of 95 men who participated voluntarily in the study: 27 IPV perpetrators who were heavy drinkers (see definition below), 33 IPV perpetrators who were not heavy drinkers, and 35 non-violent men with no history of violence as the control group. The IPV perpetrators were recruited from the community psychological and psychoeducational treatment program, CONTEXTO, carried out in the Department of Social Psychology of the University of Valencia (Spain). This is a court-mandated program for men who were sentenced to less than 2 years in prison for violence against women in intimate relationships, but had no previous criminal record, and therefore, had received a suspended sentence on the condition that they attend this intervention program [36,37,38].

Initial inclusion criteria for IPV perpetrators were as follows: having been sentenced to prison for IPV (less than 2 years in prison); not having been convicted of assault outside the home, in order to analyze the specific profile of IPV perpetrators (excluding those IPV perpetrators who presented generalized aggression); and not being diagnosed with any mental illness. Candidates continued to be eligible to participate if the qualitative interviews and Symptom Checklist-90-Revised (SCL-90-R) scores confirmed that they were free of mental illness. Use of alcohol, tobacco, and other drugs (cannabis, MDMA, heroin, and cocaine) was also registered. We then included IPV perpetrators who reported an alcohol intake of 30 g/day or more [39,40,41] and had four or more symptoms of alcohol use disorder (AUD) listed in the DSM-5 [42], forming the group of high alcohol users (HAs). In addition, those who reported an intake of less than 30 g/day and had less than two DSM-5 symptoms of AUD were classified as low alcohol users (LAs), whereas other IPV perpetrators were excluded. Moreover, we also checked that the participants (IPV perpetrators and controls) did not abuse other drugs, such as cannabis, MDMA, heroin, and cocaine, and presented less than two DSM-5 symptoms of substance use disorder (SUD).

Controls were recruited through mailings and advertisements. Inclusion criteria were as follows: having similar socio-demographic characteristics to the experimental groups (no significant differences in age, nationality, marital status, level of education, employment status, or income level; Table 1), alcohol consumption of less than 30 g/day and less than two DSM-5 symptoms of AUD, and not having been convicted of IPV, verified by a criminal record certificate attesting to the fact that they had no history of violence. Moreover, it has been suggested that scores equal to or above 11 on the conflict tactics scale-2 (CTS-2) are indicators of IPV, despite never having been convicted (Cohen et al. [43]). The control group included in our study reported lower CTS-2 scores on psychological abuse (1.50 ± 1.38), physical assault (0.13 ± 0.43), and sexual abuse (0.83 ± 1.05).

All participants were right-handed and healthy, lived in Valencia (Spain), were properly informed about the research protocol, and gave their written informed consent. The research was conducted taking into account current ethical and legal guidelines on the protection of personal data and research with human beings, in accordance with the Declaration of Helsinki, and it was approved by the Ethics Committee of the University of Valencia (H1348835571691).

### 2.2. Procedure

All participants attended three consecutive sessions at the Faculty of Psychology of the University of Valencia. In the first session, participants were interviewed to exclude those with organic diseases, and socio-demographic data were collected through a semi-structured interview. Then, participants were asked about their consumption of alcohol, tobacco, and other drugs (cannabis, MDMA, heroin, and cocaine). Subsequently, they completed an inventory based on the DSM-5 to check for the presence of AUD, and the Fagerström Test of Nicotine Dependence to assess their addiction level.

In the second session, participants carried out the laboratory cognitive task, which consists of a set of traditional neuropsychological tests and the computer-based Cambridge Neuropsychological Test Automated Battery (CANTAB). During the entire session, which lasted approximately 60 min, electrodermal activity and cardiorespiratory system activity were continuously recorded with the Vrije Universiteit Ambulatory Monitoring System (VU-AMS), using the corresponding Data Analysis and Management Software (DAMS). For later analyses, the recordings were divided into four periods: resting, preparatory, task, and recovery. In each period, the following were measured: skin conductance level (SCL), HR, respiratory rate (RR), pre-ejection period (PEP), the high frequency component (HF) of heart rate variability, and respiratory sinus arrhythmia (RSA). In addition, pre- and post-session assessments were carried out using the Positive and Negative Affect Schedule (PANAS).

In the third session, a battery of psychological trait variables were assessed using the Frontal Systems Behavior Scale (FrSBe) and Plutchik’s Impulsivity Scale. At the end of this session, participants were paid €50 for their participation.

### 2.3. Electrodermal and Cardiorespiratory Recording

The VU-AMS used to record physiological data requires seven electrodes. As recommended by the developers of the system, we used a Kendall ARBO H98SG (Covidien products, Dublin, Ireland) single use electrocardiography (ECG) electrode with Wet Gel for the impedance cardiography and ECG, and the Biopac TSD203 combined with isotonic electrode gel (GEL101) for skin conductance, which was recorded from the medial phalanges of the index and middle or ring finger. A blue lead wire connector with seven lead wires and a yellow connector were used for the recording of the ECG and SCL, respectively. An infrared interface cable connected the ambulatory recording device (VU-AMS*5fs*) to the monitoring computer. For memory, we used a 4-GB Ultra Compact Flash external memory card from SanDisk (SDCFHS-004G-G46) and a compact flash card reader to extract the VU-AMS data from the Compact Flash card. Lastly, the Data Analysis and Management Software (DAMS) was used for VU-AMS device configuration and data manipulation.

The markers used to assess ANS activity were SCL, habitually used as the main marker for emotional arousal; HR in beats per minute (bpm) and RR in breaths per minute (breath/pm) as two general physiological activation markers; the PEP index of contractility measured in milliseconds (msec) as a marker of sympathetic activity; and, finally, two markers of parasympathetic activity, namely, the HF power as a component of the heart rate variability signal (equivalent to the 0.15-0.40 Hz band) and the RSA value measured in milliseconds (msec) [44,45,46].

### 2.4. Psychological Measures

Positive and Negative Affect Schedule (PANAS): this is a self-report questionnaire composed of two scales: positive and negative affect. Each subscale is composed of 10 items that participants respond to according to how they feel at the time of the assessment. Items are rated on a Likert scale from 1 (not at all) to 5 (very much) [47,48]. Cronbach’s alpha was 0.78 for the positive affect scale and 0.82 for the negative affect scale.

Frontal Systems Behavior Scale (FrSBe): this is a 46-item behavior rating scale that was developed as a measure of behavior associated with damage to the frontal system of the brain. Index scores assess executive dysfunction, disinhibition/emotional dysregulation, and apathy. Participants rated their behaviors on a 5-point Likert-type scale. In this study, we used the Spanish version of the FrSBe [49,50]. Cronbach’s alpha was 0.84.

Plutchik’s Impulsivity Scale: impulsivity traits were assessed using the Spanish version of Plutchik’s Impulsivity Scale [51,52]. This scale is composed of 15 items rated on a Likert-type scale with four response options (never, sometimes, often, and almost always), scored from 0 to 3 (respectively). It is possible to calculate four subscales: self-control, planning, physiological behavior control, and spontaneous attitudes. Cronbach’s alpha was 0.67.

### 2.5. Data Analysis

The normality of the data distribution was explored using the Shapiro-Wilk test. After confirming the normality of the data, analysis of variance (ANOVA) was carried out to detect significant differences between groups in age, body mass index, number of children, age of starting alcohol consumption, abstinence time, nicotine consumption, nicotine dependence, criminal record for reasons other than IPV, length of sentence, personal satisfaction, internal and external locus of control, the participant’s cooperation, frustration tolerance, and questionnaire scores. In addition, chi-square tests were performed for categorical variables such as socio-demographic characteristics (nationality, marital status, level of education, employment status, etc.).

To examine group effects on psychological and physiological variables, a repeated-measures ANOVA was conducted with ‘period’ as the within-participant factor (at two time points in the case of psychological variables: pre-session and post-session; and at four time points for the physiological variables: resting, preparatory, task and recovery) and ‘group’ as the between-participant factor. The Greenhouse-Geisser correction for degrees of freedom was applied where appropriate. For significant results, partial eta-squared was reported as a measure of effect size (*η_p_*^2^). Based on a previous study, a partial eta-squared of around 0.01 was considered a small effect, around 0.06 a medium effect, and greater than 0.14 a large effect [53]. 

The areas under the curve with respect to the increase (AUCi) and ground (AUCg) were calculated using the trapezoidal formula [54] to analyze the magnitude of the responses to the task in electrodermal and cardiorespiratory variables. The AUCi was calculated with reference to the resting value, ignoring the distance from zero for all measurements and emphasizing changes over time, whereas the AUCg, the total area under the curve, was used to assess the distance of these measurements from the ground. Univariate ANOVA was used to examine group effects in AUCi and AUCg, and the Bonferroni post hoc test was then employed to determine the direction of the differences between the groups.

Data analyses were carried out using IBM SPSS Statistics for Windows, Version 22.0 (Armonk, NY, USA). In this study, *p* values < 0.05 were considered statistically significant. Average values are reported in tables as mean ± SD.

## 3. Results

### 3.1. Participants’ Characteristics

Groups did not differ in anthropometric or socio-demographic characteristics (see Table 1). Participants differed on their rates of alcohol use (see Table 1), but they did not differ on the use of other drugs, such as cannabis, MDMA, heroin, and cocaine. Furthermore, no differences were found in psychophysiological parameters measured during the resting period. Nevertheless, there were significant differences in the FrSBe total score (*F*(2, 92) = 3.36, *p* = 0.04, *η*^2^_p_ = 0.086), executive dysfunction (*F*(2, 92) = 4.53, *p* = 0.014, *η*^2^_p_ = 0.086), and disinhibition (*F*(2, 92) = 4.64, *p* = 0.012, *η*^2^_p_ = 0.086), with IPV perpetrators with high alcohol use obtaining higher scores than controls (*p* < 0.05). Moreover, there were differences in impulsivity (physiological behavior control) (*F*(2, 92) = 3.89, *p* = 0.024, *η*^2^_p_ = 0.086) and impulsivity (planning skills) (*F*(2, 92) = 15.96, *p* < 0.01, *η*^2^_p_ = 0.241), with IPV perpetrators with high alcohol use scoring lower on physiological behavior control and planning skills than controls (*p* < 0.05).

### 3.2. Stress Responses

#### 3.2.1. Psychological State Profiles and Appraisal Scores

Significant ‘period’ effects were found for PANAS positive and negative affect (*F*(1, 93) = 13.28, *p* < 0.01, *η_p_*^2^ = 0.125 and *F*(1, 93) = 9.71, *p* = 0.002, *η_p_*^2^ = 0.095, respectively), with all groups showing large decreases in their positive scores and increases in negative scores after the stressor ended (*p* > 0.05). Nevertheless, a significant ‘period x group’ interaction effect was only found for PANAS positive affect (*F*(2, 91) = 3.47, *p* = 0.035, *η_p_*^2^ = 0.071), with both groups of IPV perpetrators showing larger decreases than controls, although these differences were not significant.

Regarding appraisal, differences were observed in satisfaction (*F*(2, 88) = 16.41, *p* = 0.005, *η*^2^*_p_* = 0.270) as well as in the internal and external locus of control (*F*(2, 88) = 5.64, *p* = 0.005, *η*^2^*_p_* = 0.126 and *F*(2, 88) = 5.64, *p* = 0.005, *η*^2^*_p_* = 0.126, respectively), with both groups of IPV perpetrators obtaining lower satisfaction scores (*p* < 0.001 in both cases) and higher external locus of control scores than controls (*p* < 0.05 in both cases). Moreover, the groups differed in the evaluator’s perception of the participants’ cooperation (*F*(2, 88) = 6.00, *p* = 0.004, *η*^2^*_p_* = 0.125) and frustration tolerance (*F*(2, 88) = 10.10, *p* < 0.001, *η*^2^*_p_* = 0.219). Both groups of IPV perpetrators (HA and LA) obtained lower scores on cooperation (*p* = 0.006) and tolerance to frustration than controls (*p* < 0.001) (see Table 2).

#### 3.2.2. Electrodermal and Cardiorespiratory Responses

The cognitive task carried out in this study was effective in eliciting electrodermal and cardiorespiratory responses because significant effects of ‘period’ on the SCL, RR, HR, PEP, HF, and RSA were found in the total sample: ɛ = 0.61, *F*(1.82, 168.05) = 22.96, *p* < 0.001, *η_p_*^2^ = 0.20, β = 1; ɛ = 0.69, *F*(2.08, 191.71) = 85.56, *p* < 0.001, *η_p_*^2^ = 0.48, β = 1; ɛ = 0.94, *F*(2.82, 265.20) = 5.97, *p* = 0.001, *η_p_*^2^ = 0.06, β = 0.94; ɛ = 0.70, *F*(2.09, 197.34) = 110.15, *p* < 0.001, *η_p_*^2^ = 0.54, β = 1; and ɛ = 0.77, *F*(2.33, 214.43) = 13.76, *p* < 0.001, *η_p_*^2^ = 0.13, β = 0.99, respectively. Analyzing each group separately, intra-group comparisons revealed significant effects of ‘period’ in LA IPV perpetrators on SCL: ɛ = 0.48, *F*(1.44, 37.51) = 12.14, *p* < 0.001, *η_p_*^2^ = 0.32, β = 0.97; HR, ɛ = 0.83, *F*(2.50, 65.24) = 24.38, *p* < 0.001, *η_p_*^2^ = 0.48, β = 1; RR, ɛ = 0.61, *F*(1.83, 264.46) = 3.89, *p* = 0.029, *η_p_*^2^ = 0.10, β = 0.66; and PEP, ɛ = 0.96, *F*(2.88, 92.15) = 3.63, *p* = 0.017, *η_p_*^2^ = 0.10, β = 0.77, respectively. Moreover, in HA IPV perpetrators and controls, there was a significant ‘period’ effect on: SCL, ɛ = 0.65, *F*(1.97, 63.22) = 10.04, *p* < 0.001, *η_p_*^2^ = 0.24, β = 0.98, and ɛ = 0.41, *F*(1.23, 42.03) = 4.09, *p* = 0.041, *η_p_*^2^ = 0.10, β = 0.83, respectively; HR, ɛ = 0.59, *F*(1.78, 57.24) = 28.78, *p* < 0.001, *η_p_*^2^ = 0.47, β = 1, and ɛ = 0.62, *F*(1.86, 63.44) = 35.08, *p* = 0.041, *η_p_*^2^ = 0.50, β = 1, respectively; HF, ɛ = 0.74, *F*(2.22, 71.20) = 25.10, *p* < 0.001, *η_p_*^2^ = 0.44, β = 1, and ɛ = 0.77, *F*(2.32, 78.88) = 49.65, *p* < 0.001, *η_p_*^2^ = 0.59, β = 1, respectively; and RSA, ɛ = 0.88, *F*(2.66, 69.32) = 7.28, *p* < 0.001, *η_p_*^2^ = 0.21, β = 0.96, and ɛ = 0.84, *F*(2.52, 85.80) = 6.08, *p* = 0.002, *η_p_*^2^ = 0.15, β = 0.92, respectively. In all the groups, SCL, HR, and RR increased from resting to the preparatory period, and from the preparatory period to the tasks, then decreased until recovery. Moreover, in all groups, the PEP shortened from resting to the task period, and then lengthened until recovery. Conversely, parasympathetic markers (HF and RSA) decreased from resting to the tasks and then increased until recovery.

#### 3.2.3. Differences between Groups in Electrodermal and Cardiorespiratory Variables in Response to a Set of Cognitive Tests

**Figure 1:**
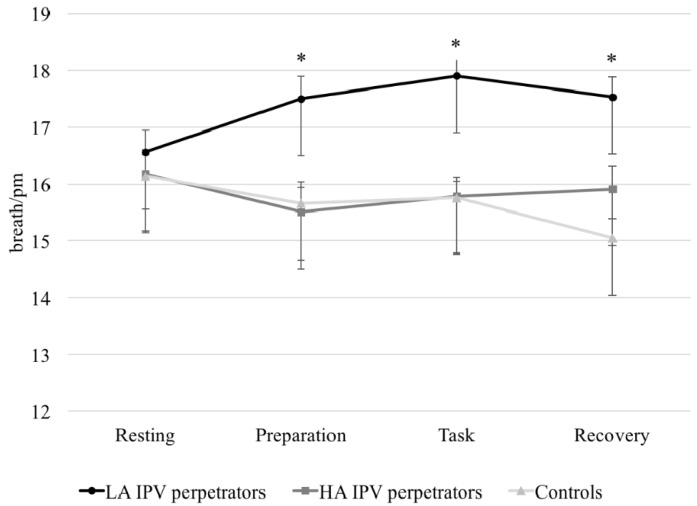
Respiratory rate (RR) (breath//pm) in response to acute stress (with different levels of alcohol consumption) and controls (* *p* < 0.05). LA: low alcohol; HA: high alcohol.

A significant ‘period × group’ interaction was found for SCL (*F*(4.18, 192.29) = 2.09, *p* = 0.05, *η*_p_^2^ = 0.44, *β* = 0.75) and RR (*F*(5.19, 238.84) = 3.86, *p* = 0.002, *η*_p_^2^ = 0.07, *β* = 0.95). In fact, LA IPV perpetrators scored higher than controls during the preparatory and recovery periods (*p* < 0.05 in both cases). Additionally, LA IPV perpetrators presented higher RR values during the preparatory, task, and recovery periods than HA IPV perpetrators and controls (*p* < 0.001 in all cases) (see Figure 1). 

**Figure 2:**
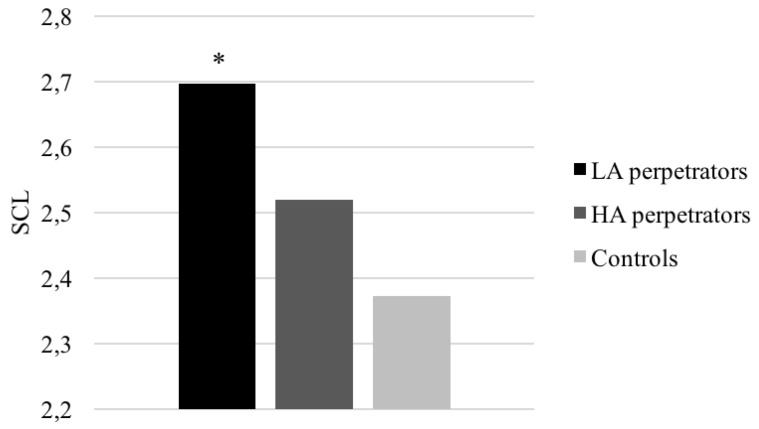
Skin conductance level (SCL) average in response to acute stress for IPV perpetrators (with different levels of alcohol consumption) and controls (* *p* < 0.05).

Finally, there was a significant ‘group’ effect for SCL (*F* (2, 92) = 3.62, *p* = 0.030, *η_p_*^2^ = 0.07, *β* = 0.66), and RR (*F*(2, 92) = 11.49, *p* < 0.001, *η_p_*^2^ = 0.20, β = 0.99), with LA IPV perpetrators showing higher SCL and RR than controls (*p* = 0.025 and *p* < 0.001, respectively). Moreover, differences were found between groups in the AUCg for SCL (*F*(2, 92) = 3.51, *p* = 0.034, *η_p_*^2^ = 0.07, *β* = 0.64) and RR (*F*(2, 92) = 9.75, *p* < 0.001, *η_p_*^2^ = 0.27, *β* = 0.65), with LA IPV perpetrators showing higher values than controls (*p* = 0.029 and *p* = 0.030). Furthermore, LA IPV perpetrators also had a higher RR AUCg than HA IPV perpetrators (*p* < 0.001) (see Figure 2).

No significant effects of ‘period × group’ or ‘group’ were observed for HR, PEP, HF, or RSA. Furthermore, there were no differences in the AUCi or AUCg in these variables.

## 4. Discussion

The aim of this study was to analyze the profile and psychological (state) and ANS (electrodermal and cardiorespiratory) response to a set of cognitive tests in two groups of IPV perpetrators with different levels of alcohol consumption, compared to non-violent individuals (controls). The present study found that IPV perpetrators (both groups) scored higher on self-reported executive dysfunctions and impulsivity (poor self-control, planning abilities, and physiological behavior control) than controls. Additionally, both groups of IPV perpetrators showed larger decreases in positive affect, less satisfaction, and a higher external locus of control than participants in the control group after the tasks ended. Regarding the psychophysiological variables, our data also demonstrated that LA IPV perpetrators presented higher SCL and RR reactivity than controls, especially during preparatory, task, and recovery periods. Nevertheless, no differences were found between groups in HR, RSA, or PEP. Finally, it should be noted that the majority of the differences between groups presented a moderate to large effect size.

The laboratory task, which can be considered a cognitive stressor and has previously been validated in clinical and normative populations employing hormonal, immunological, and psychophysiological parameters [34,35], proved to be effective in modifying emotionality and the psychophysiological state in our study. All the participants showed a significant decrease in positive affect, increases in SCL, HR, and RR, and shortening of the PEP from resting to the task periods. Furthermore, the finding of a preparatory increase in psychophysiological parameters replicates the results of previous research in which participants were confronted with different laboratory tasks involving auditory or gustatory stimuli or recognition of human faces [13]. The preparatory period is associated with increases in sympathetic activation (shorter PEP values), and this is normally followed by a decrease until the recovery period and increases in parasympathetic activation (higher HF and RSA values) [55], as we found in both IPV perpetrator groups and the non-violent controls. On the other hand, the pattern for coping with stress differed between violent and non-violent groups, but without differences between IPV perpetrators according to alcohol intake. In fact, all the IPV perpetrators rated their cognitive performance in front of a committee more negatively than controls (although we did not offer real feedback on their performance). Moreover, they attributed their performance to external factors, unlike controls, who assumed that they had control over their performance on the laboratory tasks. These results in IPV perpetrators may reflect low self-esteem and insecurity. Thus, this different way of coping with stress (different attribution) may offer an explanation for the impact of novelty on psychophysiological regulation in IPV perpetrators. However, psychophysiological differences were marked in LA IPV perpetrators and controls, but not in HA IPV perpetrators. Below, we will discuss a possible explanation for these differences or lack of them.

We initially hypothesized that reactive IPV perpetrators would show a sympathetic predominance and lower vagal activation in response to stress, especially individuals with lower alcohol consumption [5,6,26]. Specifically, impulsive IPV perpetrators tend to be characterized by an ‘electrodermal lability’, which entails sustained sympathetic activation (shorter PEP and lower vagal values), even when the stressor has ended [5,6,13]. Even though IPV perpetrators had higher self-reported executive deficits and impulsivity traits than controls (with no differences between IPV perpetrators with different levels of alcohol consumption), our data did not support the idea of a sympathetic predominance in impulsive IPV perpetrators. A possible reason for the lack of differences between groups in psychophysiological parameters could be the stressor employed. Previous research [5,6] employed a psychosocial stressor (TSST) with an emotionally charged topic for IPV perpetrators. However, the present study employed a purely cognitive stressor that is not designed to activate an emotional response in any particular group and does not interfere with or explain abnormal psychophysiological activation in IPV perpetrators. Thus, our study indicates that it would be necessary to conduct additional studies that present IPV perpetrators with different types of stimuli in order to discover whether this type of aggressor demonstrates a different/specific pattern of psychophysiological activation, or whether the activation depends on the stimulus/stressor presented.

Romero-Martínez et al. [6] concluded that impulsive IPV perpetrators showed higher general activation/arousal (HR and NSCR values) during the recovery period, but differences were only observed between LA IPV perpetrators and controls in breathing intervals (RR). Because both HR and RR may contribute considerably to HRV regulation, and there is complex feedback between the two parameters [56,57], we think our results partially agree with previous research. Moreover, our data support the view that arousal is heightened in impulsive individuals [58] because LA IPV perpetrators, who scored higher on impulsivity traits, presented higher SCL than controls.

Regarding the effects of alcohol on psychophysiological activation, our study found higher sympathetic activation in LA IPV perpetrators during the task and recovery periods than during the resting period, and this pattern was not found in HA IPV perpetrators or controls. In addition, higher activation of the parasympathetic system was found in the recovery period than in the preparatory period only in the HA group and controls. These results are partially congruent with the hypothesis that alcohol plays a core role in IPV perpetration [14,59,60,61], with alcohol consumption buffering the ANS response in this group of violent men with high alcohol consumption. Alcohol acts as a depressor of the ANS, but it is related to heightened sympathetic activation in individuals who present low alcohol consumption. Furthermore, we employed a neutral laboratory stressor without a clear emotional valence directly associated with IPV stimuli, unlike previous studies. Nevertheless, although our study showed higher activation of LA IPV perpetrators, specifically during the recovery period when the stressor had ended, it did not offer certainty about how alcohol disrupts ANS regulation, thus predisposing the individual to aggressive behavior. Tentative explanations for the lack of significant results can be provided. First, the criterion employed to classify the sample, namely, alcohol abuse, although previously employed and validated [39,40,41], has not been used in previous research on IPV perpetrators. Second, there is no clear understanding of what amount of alcohol or how many years of sustained alcohol consumption are necessary to disrupt ANS regulation [27,28,29,62]. Moreover, it is not clear whether ANS disruptions can be exclusively explained by acute alcohol consumption, rather than chronic use, without consumption during the research conducted. Additionally, future research should consider specific personality traits, such as antisocial, borderline, narcissistic, and dependent traits, which tend to present a direct association with alcohol misuse [59,63,64], in order to study how they affect the ANS response to stress. Finally, our sample is relatively young, and the participants had not been clinically diagnosed with AUD. Overall, further research is needed to clarify the ANS disruption associated with alcohol consumption, and whether IPV perpetrators present a different pattern of ANS activation when faced with stimuli with different emotional valences.

This study is part of an ongoing research effort to improve our understanding of why IPV perpetrators use violence against their partners. Even though the present study provides important information that improves our understanding of factors predisposing men to IPV, several limitations should be recognized. First, the modest sample size and the cross-sectional nature of our study could make it difficult to generalize the results obtained. Hence, further studies should be performed with a larger sample size and including other types of IPV perpetrators, such as those with generalized aggression and/or other types of antisocial behaviors, to find out whether our results can be replicated. Another limitation is the absence of a non-violent alcoholic control group. However, it is quite difficult to identify a group of alcoholic men who are still consuming alcohol and agree to voluntarily participate in research. Finally, the fact that the control group is IPV-free could not be verified because we do not have their partners’ reports. Nonetheless, our data are novel because no studies have examined electrodermal and cardiorespiratory responses to an acute laboratory stressor in IPV perpetrators.

## 5. Conclusions

The present study extends previous psychophysiological research in this field, allowing to us to extend our knowledge about how perpetrators’ ANS reacts to different stressful situations. This study was conducted as an effort to simulate daily life situations (marital conflict, psychosocial stress, cognitive stress, etc.) and understand how IPV perpetrators cope with acute stress, with the aim of developing specific interventions to improve their self-regulation. Even though we are in the early stages of developing this type of rehabilitation strategy, neurofeedback seems to offer the possibility to reduce impulsivity and improve behavioral inhibition. Moreover, the analysis of these psychobiological variables, along with neuropsychological assessments, could be used to define perpetrator typologies, which, in turn, would make it possible to develop more specific prevention and intervention programs [63,64,65,66,67,68]. Hence, in-depth knowledge about ANS regulation in IPV perpetrators could help to develop methods to use as adjuvants to current psychotherapy. For example, neurofeedback training could be used to increase batterers’ behavioral self-regulation and, in turn, increase adherence to rehabilitation interventions and reduce the risk of IPV recidivism in the long term.

## Figures and Tables

**Table 1:** Mean ± SD of descriptive characteristics for all groups (* *p* < 0.05).

Variable	IPV Perpetrators		Control (*n* = 35)	*η_p_* ^2^
	High Alcohol	Low Alcohol		
	(*n* = 27)	(*n* = 33)		
Age (years)	40.07 ± 12.10	39.84 ± 10.09	42.14 ± 10.94	0.080
Body mass index (BMI) (kg/m^2^)	22.44 ± 3.80	24.15 ± 3.41	24.46 ± 4.74	0.043
Nationality				0.079
Spanish	22 (81.48%)	26 (78.78%)	28 (80%)	
Latin Americans	3 (11.11%)	3 (9.09%)	5 (14.26%)	
Africans	2 (7.41%)	4 (12.13%)	0 (0%)	
Marital status				0.086
Single	10 (37.03%)	11 (33.33%)	16 (45.71%)	
Married	5 (18.52%)	9 (27.28%)	14 (40.00%)	
Separate/Divorced/Widowed	12 (44.45%)	13 (39.39%)	5 (14.28%)	
Number of children	0.80 ± 1.30	1.67 ± 2.08	0.86 ± 0.97	0.048
Level of education				
Primary/lower secondary	20 (70.07%)	16 (48.48%)	14 (40%)	
Upper secondary/vocational training	6 (22.22%)	15 (45.45%)	18 (51.43%)	
University	1 (2.70%)	2 (6.07%)	3 (8.57%)	
Employment status				0.065
Employed	12 (44.50%)	14 (43.75%)	15 (42.86%)	
Unemployed	15 (55.50%)	19 (59.37%)	20 (57.14%)	
Income level				0.063
1800 €–12,000 €	14 (51.86%)	13 (39.39%)	21 (60%)	
>12,000 €–30,000 €	12 (44.44%)	16 (48.49%)	12 (34.28%)	
>30,000 €–90,000 €	1 (3.70%)	4 (12.12%)	2 (5.72%)	
Age at start of alcohol consumption	16.35 ± 2.16	18.10 ± 5.16	17.06 ± 3.02	0.035
Amount of alcohol consumption per day ^1,^*	64.65 ± 8.32	9.41 ± 11.15	6.23 ± 7.90	0.260
Time of alcohol abstinence (months)	0.34 ± 0.79	1.44 ± 3.40	0.69 ± 3.35	0.080
Cigarettes/day	11.74 ± 9.04	12.76 ± 10.84	8 ± 6.41	0.046
Fagerström test	3.94 ± 2.10	4.31 ± 3.59	3.36 ± 2.76	0.670
Criminal records other than IPV				0.87
No	28 (84.85%)	21 (84%)	-	
Yes	1 (3.03%)	0 (0%)	-	
Yes, but no violence	4 (12.12%)	4 (16%)	-	
Time of sentencing (months)	9.81 ± 6.52	11.90 ± 8.89	-	0.93

^1^ Differences between intimate partner violence (IPV) perpetrators with high alcohol use and IPV perpetrators with low alcohol use and between IPV perpetrators with high alcohol use and controls. Note: Percentages may not add up to exactly 100%, owing to the rounding off.

**Table 2:** Mean ± SD of psychological measures for all groups (* *p* < 0.05).

Variable	IPV Perpetrators	Low Alcohol (*n* = 33)	Control (*n* = 35)	*η_p_* ^2^
	High Alcohol			
	(*n* = 27)			
PANAS Positive affect ^1,^*				
Pre	29.22 ± 8.88	29.61 ± 6.76	28.89 ± 7.45	0.125
Post	25.81 ± 9.35	26.03 ± 8.53	28.69 ± 8.01	0.710
Negative affect ^1^				
Pre	13.59 ± 3.51	12.70 ± 3.07	12.43 ± 2.33	0.095
Post	12.19 ± 2.74	12.06 ± 2.79	11.43 ± 2.52	0.125
Appraisal				
Satisfaction ^2^	6.37 ± 1.20	6.57 ± 1.46	8.06 ± 1.11	0.270
Internal locus of control ^2^	7.22 ± 1.62	7.19 ± 1.62	8.11 ± 0.96	0.126
External locus of control ^2^	2.78 ± 1.62	2.81 ± 1.19	1.89 ± 0.96	0.126
Cooperation ^2^	4 ± 0.69	4.17 ± 0.65	4.57 ± 0.60	0.125
Frustration tolerance ^2^	3.05 ± 0.86	3.31 ± 0.76	3.89 ± 0.58	0.219
Frontal System Behavior Scale				
Executive dysfunction ^3^	43.42 ± 10.51	36.57 ± 8.11	37.81 ± 7.72	0.086
Disinhibition ^3^	41.79 ± 11.96	35.27 ± 9.85	34.34 ± 7.14	0.086
Impulsivity Scale				
Self-Control	5.30 ± 2.90	4.27 ± 2.94	5.88 ± 2.88	0.234
Planning deficits ^3^	8.74 ± 1.94	8.28 ± 2.15	5.54 ± 2.63	0.241
Physiological behaviors control ^3^	0.63 ± 0.92	0.79 ± 1.11	1.30 ± 0.91	0.086
Spontaneous attitude	3.19 ± 1.38	2.82 ± 1.86	3 ± 1.87	0.655

IPV: intimate partner violence. ^1^ Differences between pre and post scores in all three groups. ^2^ Differences between IPV perpetrators (both groups) and control. However, no differences were found between IPV perpetrators with high and low alcohol consumption. ^3^ Differences between IPV perpetrators with high alcohol and controls.
